# Sleep Disorders in Pregnant Women and Their Impact on Maternal and Fetal Outcomes: A Narrative Review

**DOI:** 10.3390/jcm15114179

**Published:** 2026-05-28

**Authors:** Francesca Miglino, Alma Barci, Arianna Degli Agostini, Valentino Remorgida, Alessandro Libretti, Libera Troìa

**Affiliations:** 1 Department of Gynecology and Obstetrics, University Hospital Maggiore della Carità, 28100 Novara, Italy; franci.miglino@gmail.com (F.M.); almabarci@hotmail.com (A.B.); arianna.degliagostini@gmail.com (A.D.A.); libera.troia@uniupo.it (L.T.); 2 School of Obstetrics and Gynecology, University of Eastern Piedmont, 28100 Novara, Italy; 3 Department of Translation Medicine, University of Eastern Piedmont, 28100 Novara, Italy

**Keywords:** sleep breathing disorders, pregnancy, obstructive sleep apnea, hypertensive disorders in pregnancy, fetal outcomes, maternal outcomes

## Abstract

**Background/Objectives**: Sleep breathing disorders (SBDs) comprise a range of conditions characterized by abnormal respiratory patterns during sleep, with obstructive sleep apnea (OSA) being the most common. During pregnancy, SBDs are of clinical relevance, as they are associated with increased maternal and neonatal morbidity. **Methods**: A structured literature search was conducted to identify relevant studies addressing sleep breathing disordered in pregnancy, including longitudinal, observational, case-control, and cross-sectional studies, as well as other reviews and meta-analyses. **Results**: Adequate sleep during pregnancy is essential for maternal health and fetal development. OSA in pregnant women is strongly associated with hypertensive disorders of pregnancy (HDPs), potentially contributing to increased long-term cardiovascular risk. In addition to hypertensive complications, OSA has been linked to gestational diabetes and postpartum depression. Untreated SBDs may also have consequences beyond pregnancy, adversely affecting fetal and neonatal outcomes. Pathophysiological mechanisms related to maternal SBDs can result in fetal growth restriction, impaired neurocognitive development, and an increased risk of preterm birth. **Conclusions**: Current evidence indicates that OSA during pregnancy is associated with elevated short- and long-term risks for both mothers and offspring. Future research should prioritize large prospective studies with standardized diagnostic criteria and outcomes, as well as pragmatic trials to assess the implementation of SBD screening in prenatal care, particularly among high-risk populations such as obese women.

## 1. Introduction

Sleep breathing disorders (SBDs) represent a spectrum of conditions characterized by abnormalities in respiratory patterns during sleep, with obstructive sleep apnea (OSA) being the most common manifestation [1]. The prevalence of SBDs increases significantly during pregnancy, particularly among obese women, where the reported rates range from 15–20% depending on diagnostic criteria and gestational age at assessment [1,2]. This striking prevalence demands careful consideration, as mounting evidence suggests SBDs may significantly impact both maternal and fetal outcomes [3].

Pregnancy induces profound physiological changes in respiratory function, including reduced functional residual capacity, increased minute ventilation, and anatomical alterations such as weight gain and fluid retention which contribute to airway collapsibility [1].

In addition to respiratory adaptations, pregnancy is characterized by significant cardiovascular and hemodynamic changes [1]. Maternal blood volume progressively increases by approximately 40–50%, accompanied by increased stroke volume and cardiac output, while heart rate rises by 10–20 beats per minute to meet the metabolic demands of both mother and fetus [1,2]. Oxygen consumption also increases during pregnancy, leading to enhanced ventilatory drive mediated largely by progesterone [1]. These physiological adaptations, together with diaphragmatic elevation caused by the enlarging uterus, contribute to reduced functional residual capacity and increased upper airway vulnerability during sleep, thereby predisposing pregnant women to sleep breathing disorders [1,2,3].

For obese pregnant women, these normal adaptations are compounded by excess adipose tissue deposition in the neck and pharyngeal structures, resulting in an increased predisposition to upper airway collapse during sleep. Additionally, the physiological hyperemia of pregnancy exacerbates upper airway narrowing through mucosal edema, while hormonal influences—particularly elevated progesterone—alter respiratory drive and arousal thresholds [4].

The pathophysiological consequences of SBDs, characterized by intermittent hypoxia, hypercapnia, sympathetic activation, and sleep fragmentation, appear particularly detrimental in the context of pregnancy. These disturbances trigger a cascade of inflammatory and oxidative stress responses that may directly compromise placental function. Intermittent hypoxia has been shown to upregulate pro-inflammatory cytokines, including interleukin-6 and tumor necrosis factor-alpha, while simultaneously reducing anti-inflammatory mediators. This inflammatory milieu, coupled with oxidative stress, promotes endothelial dysfunction and impairs trophoblast invasion—critical elements for proper placental development and function [3,5].

At the placental level, intermittent maternal hypoxia and sleep fragmentation may impair normal placental development and function through several interconnected mechanisms [3,4,5]. Recurrent hypoxia–reoxygenation cycles promote oxidative stress, systemic inflammation, and endothelial dysfunction, contributing to abnormal spiral artery remodeling and impaired uteroplacental perfusion [3,5]. Furthermore, altered angiogenic balance, characterized by increased antiangiogenic factors such as soluble fms-like tyrosine kinase-1 (sFlt-1) and reduced placental growth factor (PlGF), may contribute to the development of hypertensive disorders of pregnancy (HDPs), including preeclampsia [3,5]. Placental inflammation and vascular dysfunction may therefore represent central mechanisms linking maternal sleep breathing disorders with adverse maternal and fetal outcomes [3,5].

The clinical implications of these pathophysiological mechanisms are substantial. SBDs in pregnant women have been associated with heightened risk of gestational hypertensive disorders, including preeclampsia, with a 2–5-fold increased risk reported in different reviews and meta-analyses varying in base of gestational age at onset and OSA severity [1,6]. Gestational diabetes mellitus shows similar associations, possibly mediated through SBD-induced insulin resistance [7]. Equally concerning are associations with intrauterine growth restriction, preterm birth, and cesarean delivery, all of which contribute to increased maternal and neonatal morbidity [2].

Although several studies and systematic reviews have investigated the association between sleep breathing disorders and adverse pregnancy outcomes, important gaps remain in the current literature. Existing evidence is often heterogeneous about diagnostic criteria, timing of assessment during pregnancy, and evaluated maternal and fetal outcomes, limiting the comparability and clinical applicability of findings. Furthermore, many reviews have focused predominantly on obstructive sleep apnea alone or on isolated maternal complications, without providing an integrated overview of the underlying pathophysiological mechanisms linking maternal sleep disorders to both short- and long-term maternal and neonatal consequences. In addition, the implications of placental dysfunction, fetal programming, and long-term offspring health remain incompletely addressed. Therefore, this narrative review aims to provide a comprehensive and updated overview of sleep breathing disorders in pregnancy, integrating current evidence on physiological mechanisms, maternal and fetal outcomes, screening approaches, and therapeutic perspectives, while highlighting unresolved clinical and research challenges.

This review aimed to describe the prevalence of sleep disorders in pregnancy, examine the biological mechanisms linking SBDs to maternal and fetal outcomes, and summarize available treatment options.

## 2. Materials and Methods

The literature search was conducted using PubMed, Embase, Web of Science, and Google Scholar for studies published up to 15 February 2026. The search strategy included combinations of the following terms: “sleep breathing disordered”, “obstructive sleep apnea”, “pregnancy”, “hypertensive disorders of pregnancy”, “gestational diabetes”, “fetal outcomes”, and “maternal outcomes”. Priority was given to studies with clinical relevance, including prospective and retrospective observational studies, case-control studies, meta-analyses, systematic reviews, and clinical guidelines. Additional references were identified through manual screening of bibliographies from relevant articles. As this study was designed as a narrative review, no formal review protocol was prospectively registered.

Studies were selected based on their relevance to the objectives of the review, with particular attention to pathophysiological mechanisms, maternal and neonatal outcomes, screening approaches, and therapeutic strategies. Non-English articles, conference abstracts without sufficient data, and studies not directly related to sleep breathing disorders in pregnancy were excluded.

Studies using different approaches for SBD diagnosis were considered eligible, including polysomnography, home sleep apnea testing, validated questionnaires, and administrative database coding (ICD codes), due to the heterogeneity of the available literature. No strict gestational age restriction was applied, as studies evaluated sleep breathing disorders across different trimesters of pregnancy. Maternal comorbidities such as obesity, chronic hypertension, and diabetes were considered during interpretation of findings because of their potential confounding role in the association between SBDs and adverse pregnancy outcomes.

Given the heterogeneity of the available literature in terms of study design, diagnostic criteria, gestational timing, and evaluated maternal and fetal outcomes, a narrative review approach was considered the most appropriate methodology to provide a comprehensive and clinically integrated overview of the topic. This approach allowed the inclusion of observational studies, reviews, meta-analyses, and emerging evidence addressing pathophysiological mechanisms, screening strategies, and maternal–fetal outcomes. However, the authors acknowledge the inherent limitations of narrative reviews, including the potential for selection bias and the lack of formal quantitative synthesis.

Studies were grouped according to the modality used for SBD diagnosis, including objective sleep testing, questionnaire-based assessment, and administrative database coding.

Given the narrative nature of this review, a formal risk-of-bias assessment was not performed. Nevertheless, differences in study design, diagnostic approaches, and outcome definitions were critically considered during the interpretation of the evidence.

## 3. Evidence Summary

### 3.1. Risk Factors

Obesity during pregnancy represents an important risk factor for the development of sleep disorders. Indeed, an observational cross-sectional study published in 2022 concluded that obese pregnant women showed a higher frequency of sleep breathing disorders (SBDs), with worsening respiratory conditions and associated complications [8].

In addition to obesity, there are various factors that can affect sleep quality, such as hormonal fluctuations and physical complaints, but also psychological factors, including fear of childbirth, maternal stress and anxiety, unwanted pregnancies, hospitalization, and fetal complications such as IUGR (intrauterine growth restriction) [9,10,11].

Anxiety, which is associated with poor sleep quality, might arise from concerns about the fetus’s health, the ability to care for the baby, and changes in body image and in the relationship with the spouse [12]. All this stress can affect sleep quality by causing the hyperactivity of the hypothalamic–pituitary–adrenal axis, increasing cortisol levels, releasing arginine vasopressin and disrupting the circadian rhythm [13,14]. Moreover, this relationship is bidirectional, so that high levels of anxiety might lead to poor sleep quality and insomnia, while poor sleep quality can increase anxiety [15].

### 3.2. Screening and Treatment

Early identification of SBDs in pregnant women, particularly those with obesity, remains challenging but essential [16]. While polysomnography represents the diagnostic gold standard, its limited availability and the logistical challenges it presents for pregnant women have prompted investigation into alternative screening approaches. Questionnaires such as the Berlin Questionnaire and STOP-BANG (Body mass index, Age, Neck circumference, Gender) have shown variable performance in pregnancy, with sensitivity often compromised by the overlap between normal pregnancy symptoms and SBD manifestations [17,18]. Home sleep apnea testing offers a promising middle ground, though validation studies specifically in pregnancy remain limited. Novel approaches utilizing continuous nocturnal oximetry with automated analysis algorithms have demonstrated promising results in recent studies and may represent a practical screening solution [19].

Treatment modalities for SBDs in pregnancy mirror those in the general population, with continuous positive airway pressure (CPAP) representing the mainstay intervention. However, adherence challenges are amplified in pregnancy, with a randomized controlled trial reporting compliance rates of only 30–50% [20]. Two systematic reviews suggest that the use of CPAP treatment may reduce rates of hypertensive disorders of pregnancy and improve fetal growth parameters, though definitive evidence from randomized controlled trials is still emerging [21,22]. Positional therapy, mandibular advancement devices, and weight management strategies offer alternative or adjunctive approaches, particularly for mild to moderate disease or when CPAP is poorly tolerated [23]. Despite growing recognition of the importance of SBDs in pregnancy, significant knowledge gaps persist [24]. The optimal timing and methods for screening remain undefined, as does the threshold for intervention. The long-term implications of maternal SBDs for offspring health trajectories warrant further investigation, with preliminary data suggesting potential impacts on childhood neurodevelopmental outcomes and metabolic programming. Perhaps most critically, there remains insufficient evidence regarding the effectiveness of SBD treatment in improving pregnancy outcomes, with most data derived from small observational studies [24].

Overall, current evidence suggests that CPAP therapy may improve blood pressure control and potentially reduce adverse maternal and fetal outcomes in selected high-risk pregnant women with OSA. However, most available studies are observational or involve small sample sizes, while randomized controlled data remain limited. Furthermore, treatment adherence during pregnancy represents a major challenge that may affect therapeutic efficacy. Therefore, although CPAP remains the standard treatment for moderate-to-severe OSA during pregnancy, additional large prospective and randomized studies are required to better define its impact on clinically relevant maternal and neonatal outcomes.

### 3.3. Maternal Outcomes

Epidemiological studies have reported that SBDs are associated with adverse health outcomes including impaired glucose tolerance, type 2 diabetes, hypertension, cardiovascular events and mortality [25].

Good sleep quality during pregnancy is crucial for both fetal development and maternal health [26]. However, half of all pregnant women experience SBDs [27], which constitute a common obesity-related co-morbidity with strong associations to cardiometabolic disease [3]. Selected studies evaluating hypertensive disorders of pregnancy and gestational diabetes risk in women with obstructive sleep apnea using different diagnostic approaches are represented in Table 1.

### 3.4. Hypertensive Disorders

Hypertension disorders in women are associated with OSA [28,29]. The prevalence of HDPs (hypertensive disorders of pregnancy) increases with OSA severity [30]. This comorbidity is caused by the repetitive cycle of hypoxemia and reoxygenation, typical of OSA, which activates the sympathetic nervous system and induces oxidative stress, which is associated with endothelial dysfunction [31,32]. This endothelial dysfunction is then associated with the relationship between OSA and cardiovascular disease [33].

In the second and third trimester of pregnancy, the associated imbalance of antiangiogenic protein is contributing to the clinical manifestations of preeclampsia, including vasoconstriction, hypertension and proteinuria, as well as lower serum levels of PAPP-A (pregnancy-associated plasma protein A) [34]. Hypertensive disorders of pregnancy, including preeclampsia, impact significantly on the fetus and the mother, increasing the risk of cardiovascular diseases in women later in life [35].

Even cardiovascular diseases, such cardiomyopathy, congestive heart failure and pulmonary edema, are associated with the diagnosis of OSA [36]. Women with OSA are five times more likely to die during a pregnancy-related admission [36].

It has been proposed that OSA treatment with CPAP may contribute to the management of HDPs [37,38].

An Australian group hypothesized that sleep breathing disorders may link hypertensive disorders of pregnancy to reduced fetal movements (a marker of fetal health) and that treating these disorders could improve fetal activity during sleep.

The conclusion was that women with preeclampsia exhibited inspiratory flow limitation and a higher level of oxygen desaturation during sleep. Preeclampsia was associated with a reduction in the total number of nocturnal fetal movements compared to controls, as well as alterations in fetal movements patterns. The number of fetal hiccups was also significantly reduced in subjects with preeclampsia. Treatment with CPAP increased the number of fetal movements and hiccups [39].

In line with this, other preliminary evidence suggests that in pregnant women with hypertension and chronic snoring, using nasal CPAP during the first eight weeks of pregnancy, together with standard prenatal care, is associated with better blood pressure control and improved pregnancy outcomes [40].

However, additional prospective studies are needed to establish the role of CPAP in management and prevention of HDPs [1].

Moreover, music therapy in pregnant women with hypertension may significantly reduce negative emotions and improve sleep quality, offering clinical promotion value [41]. However, future clinical studies are necessary to confirm these findings and determine the optimal implementation strategies and duration of music therapy [41].

Finally, the relationship between SBDs and HDPs may contribute to additional long-term cardiovascular risks [42,43]. Therefore, the management of sleep disorders during pregnancy may not only mitigate adverse maternal outcomes but also reduce long-term cardiovascular risks, which continue to represent one of the leading causes of mortality in the general population.

Taken together, the available evidence supports a significant association between SBDs and hypertensive disorders of pregnancy, although most data derive from observational studies and residual confounding cannot be excluded. The consistency of findings across large cohort studies and mechanistic data nevertheless strengthens the biological plausibility of this association.

### 3.5. Gestational Diabetes

Although several observational studies suggest an association between OSA and gestational diabetes, other studies have failed to confirm this relationship, indicating that the evidence remains heterogeneous. The risk of gestational diabetes is increased among women with mild to moderate OSA in early and mid-pregnancy, and even higher among those with severe OSA (AHI: apnea–hypopnea index > 15/h; it represents the number of episodes of apnea or hypopnea that occur per hour of sleep) [44]. Indeed, sleep disturbances may cause hypoxia stress, circadian misalignment, sympathetic activation and metabolic perturbations [45,46]. These effects might contribute to alterations in endocrine functions, reduced glucose tolerance, insulin resistance and so the development of diabetes [45].

A recent Mendelian randomization (MR) analysis provided additional support for a potential causal link between excessive daytime sleepiness and sleep apnea to the development of GDM [47]. Those are potentially preventable risk factors for GDM [47]. Mendelian randomization analysis used in the study employs genetic variants as instrumental variables to infer causal relationships between exposures (sleep traits) and outcomes (adverse pregnancy events). This approach reduces confounding through the random allocation of genetic variants at conception and avoids reverse causation bias [47].

However, a small prospective study has not found any association between GDM and sleep disorders in pregnant women with a pre-pregnancy BMI below 35 and without medical comorbidities [48].

Anyway, further studies are still necessary to determine if nocturnal CPAP therapy may have an impact on the course of this comorbidity in pregnant women [1]. There are only modest data to suggest it may improve glycemic control in non-pregnant adults [49].

Overall, current evidence suggests a probable association between SBDs and gestational diabetes, particularly in women with moderate-to-severe OSA and obesity. However, the findings remain heterogeneous, likely due to differences in study populations, diagnostic methods, gestational timing of assessment, and adjustment for confounding factors such as BMI and metabolic comorbidities. While observational studies consistently suggest an association, causal relationships and the potential benefits of CPAP therapy on glycemic outcomes during pregnancy remain insufficiently established.

### 3.6. Perinatal Depression

Perinatal depression is a mood disorder that may affect women during pregnancy or within one year after childbirth. According to the Diagnostic and Statistical Manual of Mental Disorders, Fifth Edition, Text Revision (DSM-5-TR), postpartum depression is now included under the term of perinatal depression [50].

Perinatal depression is the most common complication of childbirth and various mechanisms are involved in its development, including hormonal and neurotransmitter changes, genetic factors, neuroinflammation, psychosocial factors, economic problems, lack of social postnatal support and insufficient sleep [51].

The relationship between poor quality of sleep during pregnancy and later depressive symptoms or postnatal depression was reported by prospective cohort studies [50,52].

Moreover, other studies propose that poor sleep quality in pregnancy may be a mediator between previous depression and a subsequent depressive episode. Insomnia can facilitate the recurrence of depression in psychologically vulnerable women. Indeed, women with a history of depression before or during pregnancy who exhibited persistent insomnia symptoms were at a higher risk of developing postpartum depression [53].

A prospective cohort study observed a significant association between poor sleep quality in late pregnancy and lower levels of mental well-being five weeks postpartum. Post hoc analyses revealed a significant interaction with parity, indicating that multiparous women were more strongly affected by poor sleep quality in late pregnancy. In conclusion, sleep quality in late pregnancy is associated with maternal mental health in the early postpartum period, a phenomenon that appears to be more pronounced in multiparous women [54].

Screening for perinatal depression should be a routine part of prenatal and postpartum care, according to a wide range of guidelines [55]. It can be done using tools such as the Edinburgh Postnatal Depression Scale (EPDS) to identify individuals at risk. Treatment generally involves a combination of psychotherapy, support groups, and medication, including antidepressants, which can be safely used during pregnancy and lactation [56].

### 3.7. Fetal and Neonatal Outcomes

The pathophysiological mechanisms of maternal SBDs contribute significantly to adverse fetal and neonatal outcomes through multiple interconnected pathways. Intermittent hypoxia and reoxygenation episodes characteristic of SBDs trigger placental oxidative stress and inflammation, resulting in placental insufficiency that compromises fetal oxygen and nutrient supply, potentially leading to small-for-gestational-age (SGA) infants or fetal growth restriction (FGR) [57]. Pamidi and Marc, in their prospective cohort study, evaluated whether a polysomnography-based diagnosis of an SBD in the third trimester is associated with the delivery of SGA infants. Participants were recruited as part of a multicenter pregnancy cohort study and were assessed for SBDs based on symptoms (snoring and/or witnessed apneas, evaluated using the Pittsburgh Sleep Quality Index questionnaire) as well as through in-home full polysomnography in the third trimester. Symptoms of SBDs in the third trimester showed a potential association with the delivery of SGA infants; however, this did not reach statistical significance (OR 2.36; 95% CI 0.85–6.54; *p* = 0.10). In contrast, the odds of delivering an SGA infant were significantly increased in the presence of a polysomnography-based diagnosis of a maternal SBD (using an apnea–hypopnea index cut-off of 10; OR 2.65; 95% CI 1.15–6.10; *p* = 0.02) [57,58]. Chronic intermittent hypoxia also appears to disrupt fetal neurodevelopment through alterations in cerebral oxygenation and inflammatory mediators crossing the placental barrier, potentially contributing to delayed neurocognitive development observed in infants of mothers with untreated SBDs [57,59].

Different studies have shown a higher risk of preterm birth in pregnant women with SBDs even though the mechanism has not been clarified; the systemic inflammatory state and endothelial dysfunction present in maternal SBDs might trigger a premature cervical ripening and uterine contractions or the presence of SBD-related pregnancy complications might predispose to medically indicated preterm birth from hypertensive disorders of pregnancy including preeclampsia or suspected fetal compromise [57,58,59,60,61].

Preterm birth is defined as all births delivered before 37 weeks and it can severely impact maternal and child health, since it can be associated with neonatal mortality [62,63]. There are other lifestyle factors that have been reported to increase risks of preterm birth, such as alcohol consumption, lack of physical exercise and psychosocial stress [64,65,66]. Moreover, the identified association between poor sleep quality trajectory and an increased risk of preterm birth emphasizes the imperative to identify high-risk groups as priority targets for intervention and treatment [67].

Several studies have investigated the relationship between SBD-induced hypoxia and fetal heart rate (FHR) patterns. Case reports have demonstrated a direct correlation between maternal hypoxia and alterations in fetal acid-base status accompanied by FHR decelerations [68,69]. Additionally, a prospective cohort study found that most of both late and prolonged FHR decelerations coincided with maternal respiratory events, with each unit increase in the apnea–hypopnea index associated with a 22% increase in the odds of prolonged deceleration [70].

A prospective observational study demonstrated that, among the 4 pregnant women out of 35 (11.4%) diagnosed with OSA, 3 exhibited fetal heart rate decelerations concomitant with maternal desaturation. Babies born to mothers with OSA had significantly lower mean Apgar scores and birth weights, if compared to those born to women without OSA. Furthermore, three neonates of mothers with OSA had to be admitted in the NICU (neonatal intensive care unit) [71].

However, these findings contrast with results from a prospective trial by Olivarez et al. examining OSA in pregnancy and FHR monitoring [72]. In this study, the only FHR abnormalities observed were variable decelerations considered appropriate for gestational age. This discrepancy raises important questions regarding whether fetal hypoxia occurs during maternal apneic episodes and whether it represents a primary mechanism underlying the adverse pregnancy outcomes associated with OSA. In support of this, a prospective observational cohort study reported no significant association between apneic episodes and fetal heart rate abnormalities among 20 women with an AHI exceeding 5/h [72].

Neonatal intensive care unit admissions are higher among infants born to mothers with SBDs [36,57], reflecting the cumulative impact of these pathophysiological mechanisms on neonatal adaptation and health, and there are studies suggesting a higher risk for congenital anomalies, especially musculoskeletal anomalies, in offspring of pregnancies with OSA [73].

**Table 1:** Selected studies evaluating hypertensive disorders of pregnancy and gestational diabetes risk in women with obstructive sleep apnea using different diagnostic approaches.

Authors	Study Type	Total Number of Women (N: Women With OSA; C. Without OSA)	Inclusion Criteria	Method Used for OSA/SBD Assessment	Gestational Age at Time of Sleep Testing	GHTN	HDP	Preeclampsia	GDM
**Bisson et al.** [48]	P	52	Single pregnancy; no pre-existing chronic respiratory syndrome; no pre-gestational diabetes	PSG	24–33	N/A	N/A	N/A	no association
**Spence et al.** [61]	R	118,913 (N: 57; C: 118,856)	Single pregnancy in military hospitals	PSG	late pregnancy	N/A	OR 2.46 (95% CI 1.3–4.68)	OR 2.42 (95% CI 1.43–4.09)	N/A
**Pamidi et al.** [58]	MR	234	Single pregnancy; No pre-existing diabetes or chronic hypertension	PSG	third trimester	N/A	OR 2.3 (95% CI 1.1–4.5)	N/A	not pooled
**Louis et al.** [60]	R	171	OSA in pregnancy	PSG	all trimesters	N/A	N/A	OR 2.0 (95% CI 0.8–5.0)	N/A
**Louis et al.** [2]	P	202 (N: 27; C: 175)	BMI > 30 Kg/m^2^	PSG	all trimesters	N/A	N/A	OR 3.55 (95% CI 1.12–11.3)	no association
**Facco et al.** [44]	P	751	Nulliparous; no pre-existing diabetes or chronic hypertension	PSG	16–21	N/A	N/A	N/A	OR 2.24 (95% CI 1.09–4.6)
**Facco et al.** [30]	P	3705 (N: 318; C: 3387)	Nulliparity, singleton	HST	22–31	N/A	OR 1.5 (95% CI 0.9–2.3)	OR 1.94 (95% CI 1.07–3.5)	OR 3.5 (95% CI 2.0–6.2)
**Bourjeily et al.** [73]	R	1,423,099 (N: 1739; C: 1,421,360)	OSA in pregnancy	ICD code	all trimesters	OR 1.7 (95% CI 1.4–2.0)	N/A	N/A	OR 1.5 (95% CI 1.3–1.7)
**Louis et al.** [36]	R	55,781,965 (N: 15,445; C: 55,766,520)	All pregnant women	ICD code	all trimesters	OR 1.3 (95% CI 1.1–1.5)	N/A	OR 2.5 (95% CI 2.2–2.9)	OR 1.9 (95% CI 1.7–2.1)
**Reutrakul et al.** [7]	R	169 (N: 108; C: 61)	No pre-gestational diabetes	PSQI	third trimester	N/A	N/A	N/A	OR 3.0 (95% CI 1.2–7.4)

Abbreviation: BMI, body mass index; GDM, gestational diabetes mellitus; HST, home sleep test; ICD, International Classification of Diseases; OSA, obstructive sleep apnea; P, prospective, observational; R, retrospective, population-based, cross-sectional analysis; MR, Mendelian randomization; PSG, polysomnography; GHTN, gestational hypertension; HDP, hypertensive disorders of pregnancy; PSQI, Pittsburgh Sleep Quality Index; N/A, not available.

The potential long-term consequences for offspring exposed to maternal SBDs in utero remain incompletely characterized but raise significant concerns [74].

A recent study by Salihu showed that women at higher risk of SBDs had neonates with shorter telomere lengths in their DNA obtained from cord blood samples [75]. Shorter telomere lengths are associated with accelerated aging and age-related disease but have also been observed in adults with OSA [75,76].

Recent evidence suggests that maternal SBDs may also program long-term metabolic dysfunction in offspring through epigenetic modifications, with animal models demonstrating persistent changes in adipokine gene expression and metabolic regulation in offspring exposed to gestational intermittent hypoxia [77,78], and one observational, prospective study suggesting that maternal mild SBDs during pregnancy affect the growth of head circumference and adiposity parameters during the first three years of life [79].

Emerging evidence suggests subtle neurodevelopmental differences and potential cardiometabolic program mild effects that may persist into childhood and beyond, representing a transgenerational perpetuation of adverse outcomes [57,59].

These findings align with the developmental origins of health and disease (DOHaD) hypothesis, suggesting that disruptions to the intrauterine environment may have far-reaching implications across the lifespan. Collectively, these findings underscore the critical importance of early identification and effective management of SBDs during pregnancy not only for immediate pregnancy outcomes but also for long-term maternal–child health trajectories [80,81,82,83]. Table 2 shows studies of OSAS patients and their fetal and neonatal complications.

Overall, although several studies support an association between maternal SBDs and adverse fetal and neonatal outcomes, the magnitude of these risks remains variable across studies. Differences in diagnostic criteria, severity of OSA, and population characteristics likely contribute to the heterogeneity of findings. Further prospective studies using standardized sleep assessment methods are required to better clarify causality and long-term offspring implications.

Interpretation of the available evidence requires caution due to substantial methodological heterogeneity among studies. Diagnostic approaches to sleep breathing disorders vary considerably, ranging from polysomnography and home sleep apnea testing to questionnaire-based screening tools and administrative ICD-code databases, each with different diagnostic accuracy and risk of misclassification. Furthermore, findings derive from both small perspective cohorts and large population-based studies, each presenting distinct strengths and limitations regarding phenotypic characterization, statistical power, and susceptibility to residual confounding. These methodological differences likely contribute to variability in reported associations and should be considered when interpreting the overall evidence.

### 3.8. Limitations

This narrative review should be interpreted considering several limitations. First, the available literature is highly heterogeneous regarding study design, diagnostic methods for SBDs, gestational timing of assessment, and evaluated maternal and fetal outcomes, limiting direct comparability among studies. Second, most available evidence derives from observational studies, which limits causal inference and may be influenced by residual confounding factors such as obesity, metabolic disease, and pre-existing cardiovascular risk. Furthermore, the inclusion of studies using different diagnostic approaches, including polysomnography, home sleep testing, questionnaires, and administrative database coding, may have introduced variability in diagnostic accuracy and risk of misclassification. Publication bias cannot be excluded, particularly given the tendency for studies reporting positive associations to be more frequently published. Finally, as this work was designed as a narrative review, no formal risk-of-bias assessment or quantitative synthesis were performed.

## 4. Conclusions

Emerging evidence suggests that sleep breathing disorders, particularly obstructive sleep apnea, represent an important and frequently underrecognized condition during pregnancy, especially among obese women and those with cardiometabolic risk factors. The current literature consistently supports an association between maternal SBDs and adverse maternal outcomes, including hypertensive disorders of pregnancy, gestational diabetes, cardiovascular complications, and impaired mental health, as well as adverse fetal and neonatal outcomes such as fetal growth restriction, preterm birth, and increased NICU admission rates. Although much of the available evidence remains observational and heterogeneous, the overall consistency of epidemiological findings together with the underlying biological plausibility supports the clinical relevance of SBDs in pregnancy.

From a clinical perspective, early recognition of sleep disturbances during pregnancy may represent an important opportunity to improve maternal and fetal risk stratification, particularly in high-risk populations. However, important challenges remain regarding the identification of reliable pregnancy-specific screening tools, standardized diagnostic criteria, and evidence-based management strategies.

Future research should focus on large prospective multicenter studies using standardized definitions and objective sleep assessments, as well as randomized controlled trials evaluating the impact of CPAP therapy on clinically meaningful maternal and neonatal outcomes. Further investigation into placental dysfunction, biomarkers of disease severity, long-term maternal cardiovascular consequences, and offspring neurodevelopmental and metabolic outcomes may also help clarify the broader implications of sleep breathing disorders across the maternal–fetal lifespan.

## Figures and Tables

**Table 2:** Studies evaluating fetal and neonatal outcomes associated with maternal sleep breathing disorders.

Author	Study Type	Total Number of Women (N: Women With OSA; C. Without OSA)	OSA Status of Mother	Preterm Birth (<32 Weeks)	Preterm Births (<37 Weeks)	5-min Apgar < 7	NICU Admission	Perinatal Death	LGA	SGA	LBW
**Spence et al.** [61]	R	118,913 (N: 57; C: 118.856)	PSG	N/A	OR 1.90 (95% CI 1.09–3.30)	N/A	N/A	N/A	N/A	N/A	N/A
**Louis et al.** [2]	P	161 (N: 26, C: 135)	PSG	Not reported	Not reported	N/A	OR 3.39 (1.23–9.32)	N/A	N/A	N/A	N/A
**Pamidi et al.** [58]	P	234	PSG	N/A	N/A	N/A	N/A	N/A	N/A	OR 2.36 (95% CI 0.85–6.54)	N/A
**Morrakotkhiew et al.** [59]	P	159 (N: 136; C: 23)	PSG	N/A	OR 1.59 (95% CI 1.11–2.28)	N/A	N/A	N/A	N/A	N/A	N/A
**Pitts et al.** [70]	P	48	PSG	N/A	Not reported	N/A	OR 3.27 (95% CI 0.89–12.01)	N/A	OR 1.5 (95% CI 0.23–9.86)	Not reported	N/A
**Louis et al.** [60]	R	171	PSG	N/A	OR 1.63 (95% CI 1.4–1.89)	N/A	N/A	N/A	OR 1.48 (95% CI 1.2–1.81)	OR 1.15 (95% CI 0.91–1.46)	OR 1.76 (95% CI 1.5–2.08)
**Bourjeily et al.** [73]	R	1,423,099 (N: 1739; C: 1,421,360)	ICD	N/A	OR 1.48 (95% CI 1.29–1.69)	Not reported	OR 1.92 (95% CI 1.69–2.19)	Not reported	OR 1.1 (95% CI 0.94–1.28)	OR 1.01 (95% CI 0.85–1.18)	N/A
**Louis et al.** [36]	R	55,781,965 (N: 15,445; C: 55,766,520)	ICD code	N/A	OR 1.2 (95% CI 1.1–1.4)	N/A	N/A	N/A	N/A	N/A	N/A
**Bin et al.** [80]	R	636,746 (N: 519; C: 636,227)	ICD code	N/A	OR 1.5 (95% CI 1.2–1.8)	OR 1.6 (95% 1.1–2.4)	OR 1.3 (95% CI 1.1–1.4)	OR 1.7 (95% CI 0.9–3.3)	OR 1.3 (95% CI 1.0–1.6)	OR 0.8 (95% CI 0.6–1.1)	N/A
**Louis et al.** [16]	R	55,781,965	HST	N/A	OR 1.2 (95% CI 1.1–1.4)	N/A	N/A	OR 1.0 (95% CI 0.7–1.5)	N/A	OR 1.2 (95% CI 1.0–1.5)	N/A

Abbreviations: LGA, large for gestational age; SGA, small for gestational age; LBW, low birth weight < 2500 g; OSA, obstructive sleep apnea; P, prospective, observational; R, retrospective, population-based, cross-sectional analysis; NICU, neonatal intensive care unit; C, controls; ICD, International Classification of Diseases; PSG, polysomnography. Not reported: data not provided in the original study. N/A: not applicable.

## Data Availability

Datasets are available through the corresponding author upon reasonable request.

## Abbreviations

The following abbreviations are used in this manuscript:

AHI	Apnea–Hypopnea Index
BMI	Body Mass Index
GDM	Gestational Diabetes Mellitus
HST	Home Sleep Test
ICD	International Classification of Disease
OSA	Obstructive Sleep Apnea
P	Prospective, Observational
R	Retrospective, Population-based, Cross-sectional Analysis
RCT	Randomized Controlled Trial
MR	Mendelian Randomization
PSG	Polysomnography
GHTN	Gestational Hypertension
HDP	Hypertensive Disorders of Pregnancy
LGA	Large for Gestational Age
SGA	Small for Gestational Age
LBW	Low Birth Weight < 2500 g
NICU	Neonatal Intensive Care Unit
C	Controls
